# *Moniezia benedeni* drives the SNAP-25 expression of the enteric nerves in sheep's small intestine

**DOI:** 10.1186/s12917-024-04140-6

**Published:** 2024-07-01

**Authors:** Zhen Huang, Wanling Yao, Wanhong He, Jing Pan, Wenzhu Chai, Baoshan Wang, Zhitao Jia, Xiping Fan, Wenhui Wang, Wangdong Zhang

**Affiliations:** 1 College of Veterinary Medicine, Gansu Agricultural University, Lanzhou, 730070 China; 2 People’s Government of Heisongyi Township, Wuwei, 733000 China

**Keywords:** Sheep intestine, *Moniezia benedeni* infection, SNAP-25, Prokaryotic expression

## Abstract

**Background:**

The neuroimmune network plays a crucial role in regulating mucosal immune homeostasis within the digestive tract. Synaptosome-associated protein 25 (SNAP-25) is a presynaptic membrane-binding protein that activates ILC2s, initiating the host's anti-parasitic immune response.

**Methods:**

To investigate the effect of *Moniezia benedeni* (*M. benedeni*) infection on the distribution of SNAP-25 in the sheep's small intestine, the recombinant plasmid pET-28a-SNAP-25 was constructed and expressed in BL21, yielding the recombinant protein. Then, the rabbit anti-sheep SNAP-25 polyclonal antibody was prepared and immunofluorescence staining was performed with it. The expression levels of SNAP-25 in the intestines of normal and *M. benedeni*-infected sheep were detected by ELISA.

**Results:**

The results showed that the SNAP-25 recombinant protein was 29.3 KDa, the titer of the prepared immune serum reached 1:128,000. It was demonstrated that the rabbit anti-sheep SNAP-25 polyclonal antibody could bind to the natural protein of sheep SNAP-25 specifically. The expression levels of SNAP-25 in the sheep's small intestine revealed its primary presence in the muscular layer and lamina propria, particularly around nerve fibers surrounding the intestinal glands. Average expression levels in the duodenum, jejunum, and ileum were 130.32 pg/mg, 185.71 pg/mg, and 172.68 pg/mg, respectively. Under conditions of *M. benedeni* infection, the spatial distribution of SNAP-25-expressing nerve fibers remained consistent, but its expression level in each intestine segment was increased significantly (*P *< 0.05), up to 262.02 pg/mg, 276.84 pg/mg, and 326.65 pg/mg in the duodenum, jejunum, and ileum, and it was increased by 101.06%, 49.07%, and 89.16% respectively.

**Conclusions:**

These findings suggest that *M. benedeni* could induce the SNAP-25 expression levels in sheep's intestinal nerves significantly. The results lay a foundation for further exploration of the molecular mechanism by which the gastrointestinal nerve-mucosal immune network perceives parasites in sheep.

## Background

The gut is a crucial organ in animals for the digestion and absorption of nutrients [1, 2], while also serving as a defensive barrier [3], it prevents harmful microbes and parasites within the lumen invasion and also defends against its metabolites [4, 5]. Some studies have reported that neurons and immune cells can be highly coordinated and jointly regulated to monitor and respond to potential threats from the gut lumen [6]. For instance, neuromedin U (NMU) can activate ILC2s and initiate the host's anti-parasitic immune response [7, 8], the vasoactive intestinal peptide (VIP) can promote the activation of ILC2s and ILC3s [9, 10], and to enhance the host's mucosal barrier function and resistance to parasites. Therefore, the intestinal neuroimmune network plays a pivotal role in regulating the balance of the intestinal environment.

Synaptosomal-associated Protein 25 (SNAP-25) is a key protein involved in forming the Soluble N-ethylmaleimide Sensitive Factor Attachment Protein Receptor (SNARE) complexes, it is termed a SNARE protein together with synaptic vesicle protein (VAMP) and synaptic fusion protein (Syntaxin) [11]. SNAP-25, originally identified and named by Oyler [12]et al., belongs to an evolutionarily conserved protein family. It is a presynaptic membrane-binding protein, primarily expressed on the cytoplasmic surface of the plasma membrane, and is soluble [13]. It is mainly involved in neurotransmitter release and the regulation of neuronal cell plasticity [14–16]. Other studies have found that SNAP-25 can be involved in the regulation of autophagy [17–19], insulin secretion [20, 21], cancer [22, 23], dendritic and axonal growth [24], and learning and memory [25–27], et al. SNAP-25 also plays an important role in anti-parasitic infection. For example, SNAP-25 is closely related to mediating the targeted apical ring vesicle trafficking of Toxoplasma [28]. It has a significant correlation with eosinophils in eosinophilic gastritis [29]. In the incidence of eosinophils in nasal polyps, ILC2s are distributed near the SNAP-25 expression region, which indicated that there was a morphological coexistence relationship between SNAP-25 and ILC2s [8]. And it suggests that SNAP-25 plays an important role in the ILC2s-mediated immune response involved in parasitic resistance.

*M. benedeni*, parasitizing in the small intestines of sheep, belongs to the Anoplopcephalidae family and *Moniezia* genus, and was first reported in 1944. *Scheloribates sp.* is its crucial intermediate host. It is prevalent worldwide, mostly endemic, and it is also an important pathogen of sheep tapeworm disease. Our previous research showed that *M. benedeni* infection can substantially reduce the densities of IgA^+^, IgG^+^, and IgM^+^ cells in the sheep's intestine [30], and markedly enhance the density of intestinal IgE^+^ cells [51] and CD3^+^ T cells [53] as well as the expression of NMU [31].However, at present, there are few reports regarding the impact of *M. benedeni* infection on the expression of SNAP-25 in the sheep's intestine. In this study, the prokaryotic gene expression of SNAP-25 in sheep was investigated, the rabbit anti-sheep SNAP-25 polyclonal antibodies were prepared, and the expression and distribution patterns of SNAP-25 in the sheep's intestine both before and after *M. benedeni* infection were analyzed. It would lay the foundation for further revealing the intestinal mucosal immune network of sheep perceiving the parasitism of parasites.

## Results

### Physicochemical property

The molecular formula for sheep SNAP-25 is C_2568_H_3977_N_823_O_731_S_12_, with a theoretical isoelectric point (pI) of 4.74, indicating it is an acidic protein. The predicted instability index was approximately 39.84, which is less than 40, suggesting the protein is stable. As shown in Table 1, of the 206 amino acids composing SNAP-25, glutamic acid accounted for the highest percentage (11.20%), while tryptophan and histidine both accounted for the lowest (0.50% each).

**Table 1:** Amino acid composition of SNAP-25 in sheep

Amino acid	Quantity	Proportion	Amino acid	Quantity	Proportion
Ala(A)	16	7.80%	Leu(L)	16	7.80%
Arg(R)	17	8.30%	Lys(K)	12	5.80%
Asn(N)	15	7.30%	Met(M)	13	6.30%
Asp(D)	19	9.20%	Phe(F)	2	1.00%
Cys(C)	4	1.90%	Pro(P)	2	1.00%
Gln(Q)	13	6.30%	Ser(S)	10	4.90%
Glu(E)	23	11.20%	Thr(T)	6	2.90%
Gly(G)	14	6.80%	Trp(W)	1	0.50%
His(H)	2	1.00%	Tyr(Y)	1	0.50%
Ile(I)	11	5.30%	Val(V)	9	4.40%

### Hydrophilicity/hydrophobicity, epitopes, transmembrane regions, and signal peptide prediction

**Fig. 1:**
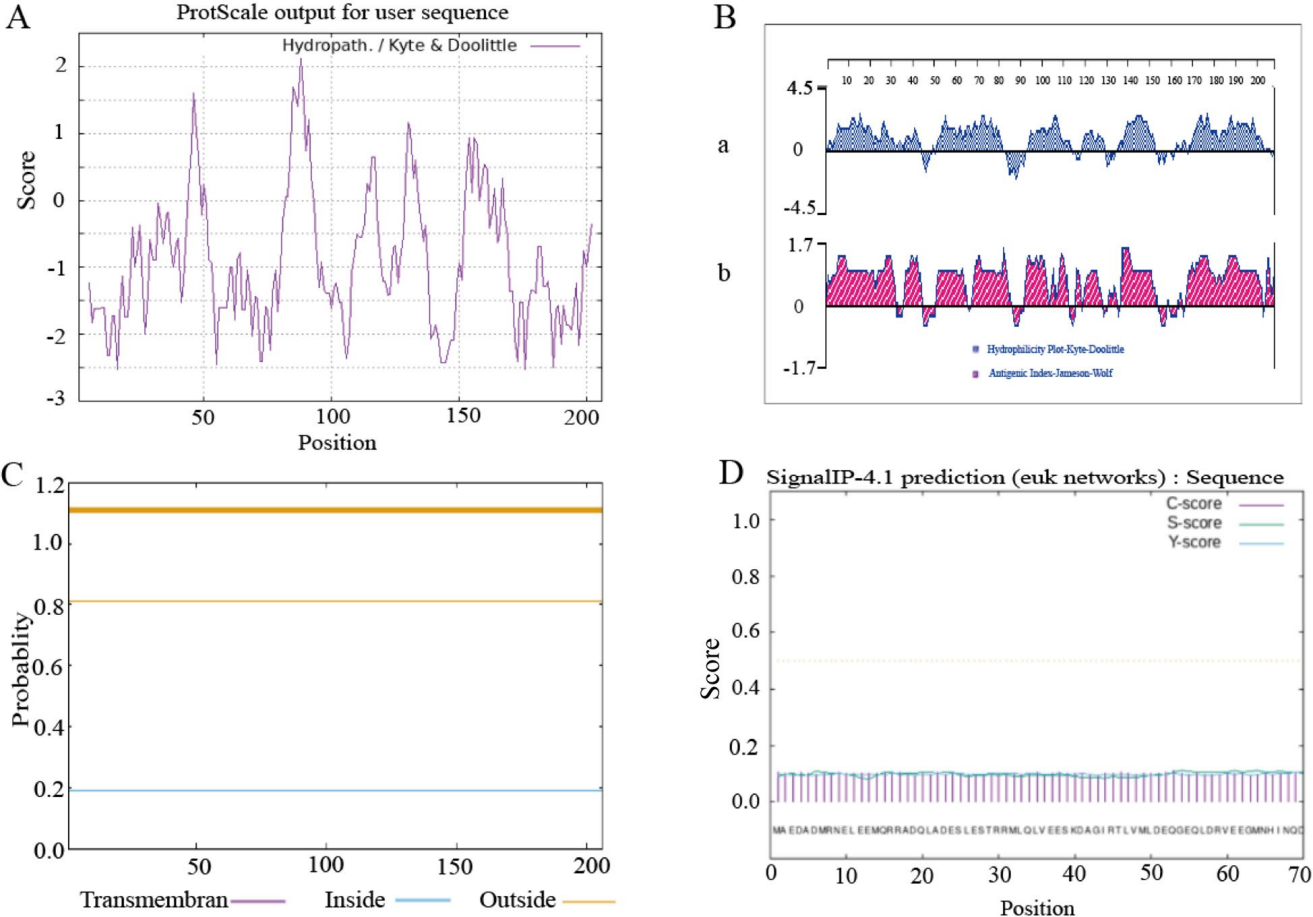
SNAP-25 proteins hydrophilic, antigen epitope, proteins across the membrane area and signal peptide prediction. **A **Hydrophobicity prediction; **B** Hydrophilicity and epitope prediction (a. hydrophilicity prediction; b. epitope prediction); **C** Transmembrane region prediction (purple line. Transmembrane region; The blue line. Within the membrane area; The yellow line. Membrane outer zone); **D **the signal peptide prediction (C-score. Splice site value; S-score. Signal peptide region value; Y-score. Parameters that integrate S and C values)

The proportion of amino acids in the hydrophilic region exceeds that in the hydrophobic region, indicating that sheep SNAP-25 is a hydrophilic protein. Analysis and prediction of hydrophilicity (Fig. 1A) and antigenic epitopes (Fig. 1B) revealed that the protein has a high antigen index. The predicted transmembrane structure showed that all amino acids of the SNAP-25 protein are located in the extracellular region (Fig. 1C); furthermore, the signal peptide prediction indicated the absence of a signal peptide segment (Fig. 1D).

### Secondary and tertiary structure prediction

**Fig. 2:**
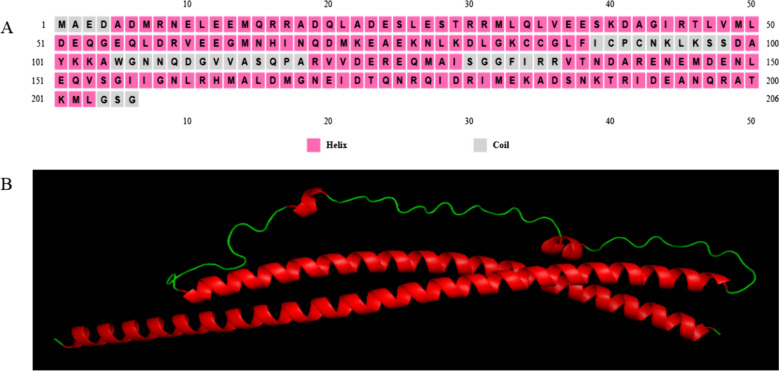
Prediction of secondary and tertiary structure of SNAP-25 protein. **A** Secondary structure prediction; **B **Tertiary structure prediction

As depicted in Fig. 2A, the α-helix region of the SNAP-25 protein constitutes the largest portion, suggesting that the SNAP-25 protein possesses intricate biological functions. The tertiary structure predictions aligned with the findings of the secondary structure (Fig. 2B).

### Phosphorylation and glycosylation sites

**Fig. 3:**
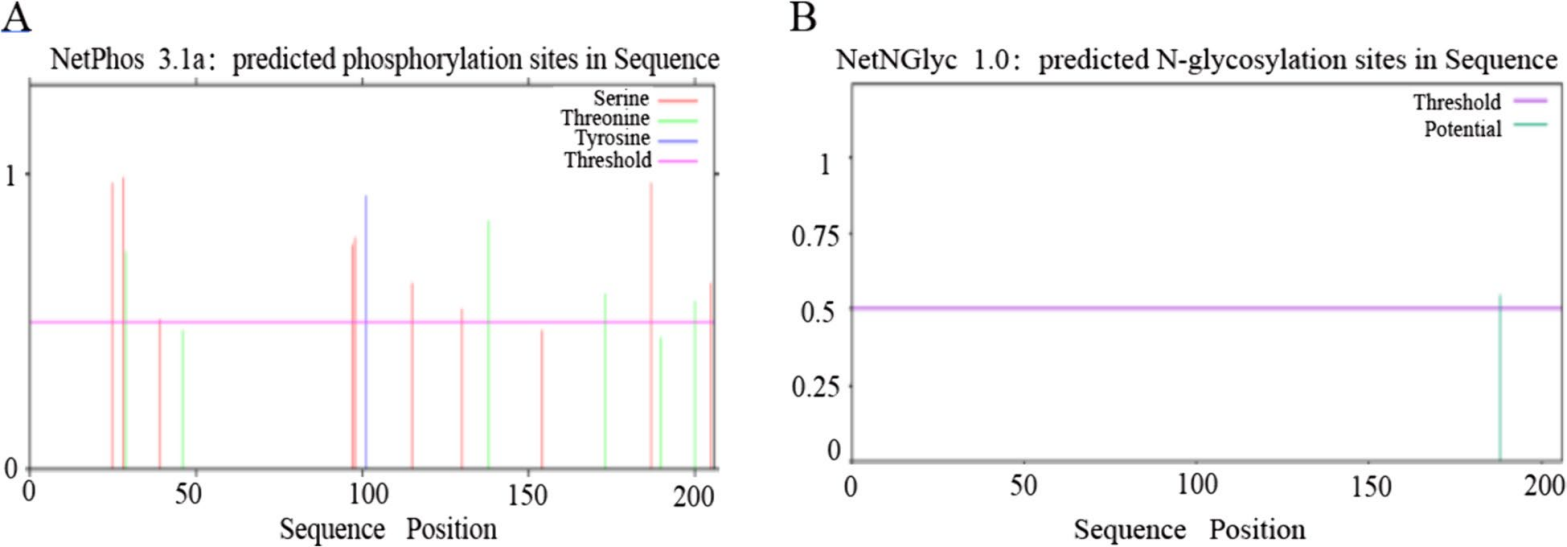
Analysis of potential phosphorylation and glycosylation sites of sheep SNAP-25 protein. **A **Analysis of potential phosphorylation sites; **B **Glycosylation sites

Phosphorylation and glycosylation sites of SNAP-25 were predicted by using NetPhos3.1 and NETNGlyc1.0 online tools. The analyses revealed the presence of 9 serines, 6 threonines, and 1 tyrosine, which are potential phosphorylation sites for protein kinases (Fig. 3A) and 1 glycosylation site (Fig. 3B), indicating that post-translational modifications of the SNAP-25 protein primarily involve phosphorylation.

### Protein interaction analysis

**Fig. 4:**
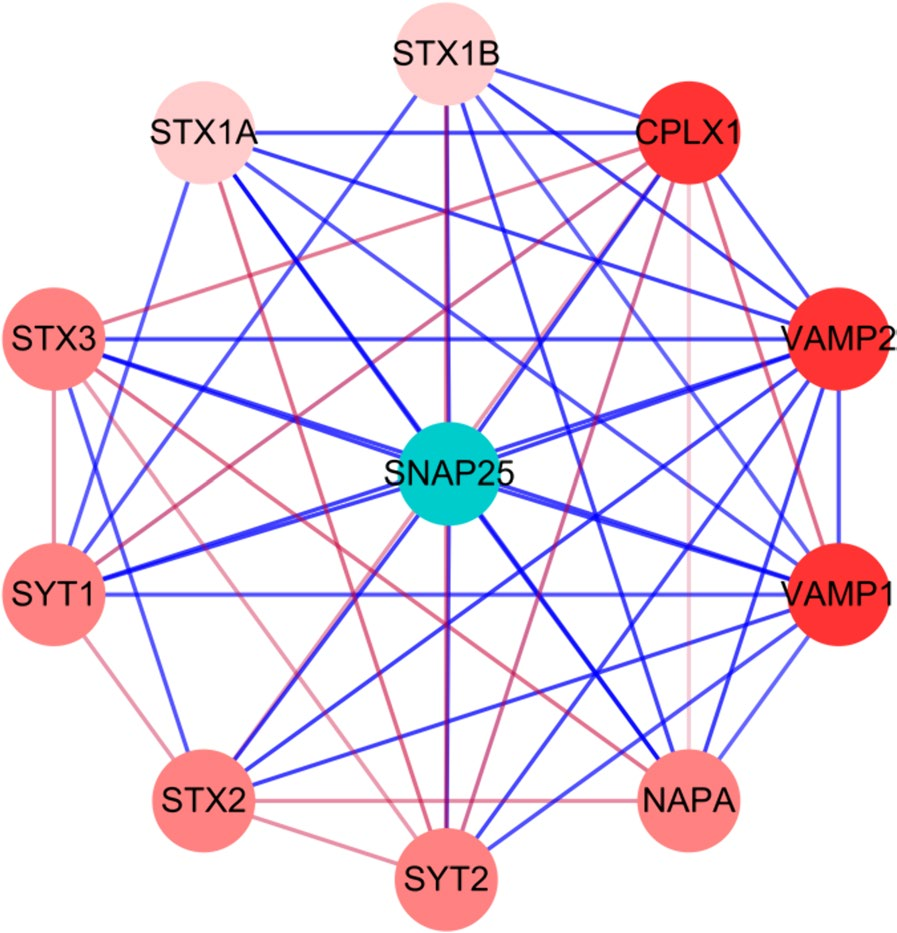
Interaction between SNAP-25 protein and other proteins in sheep. The red lines showed weak protein–protein interactions

The STRING database revealed interactions between the sheep SNAP-25 protein and synaptic vesicle protein I (SYT1), syntaxin1A (STX1A), vesicle-associated membrane protein II (VAMP2), complexin1 (CPLX1), syntaxin1B (STX1B), syntaxin3 (STX3), synaptic vesicle protein II (SYT2), syntaxin4 (STX4), and N-ethylmaleimide-sensitive factor attachment protein α (NAPA). A significant interplay was observed among these proteins (Fig. 4).

### Preparation of polyclonal antibody against sheep SNAP-25

**Fig. 5:**
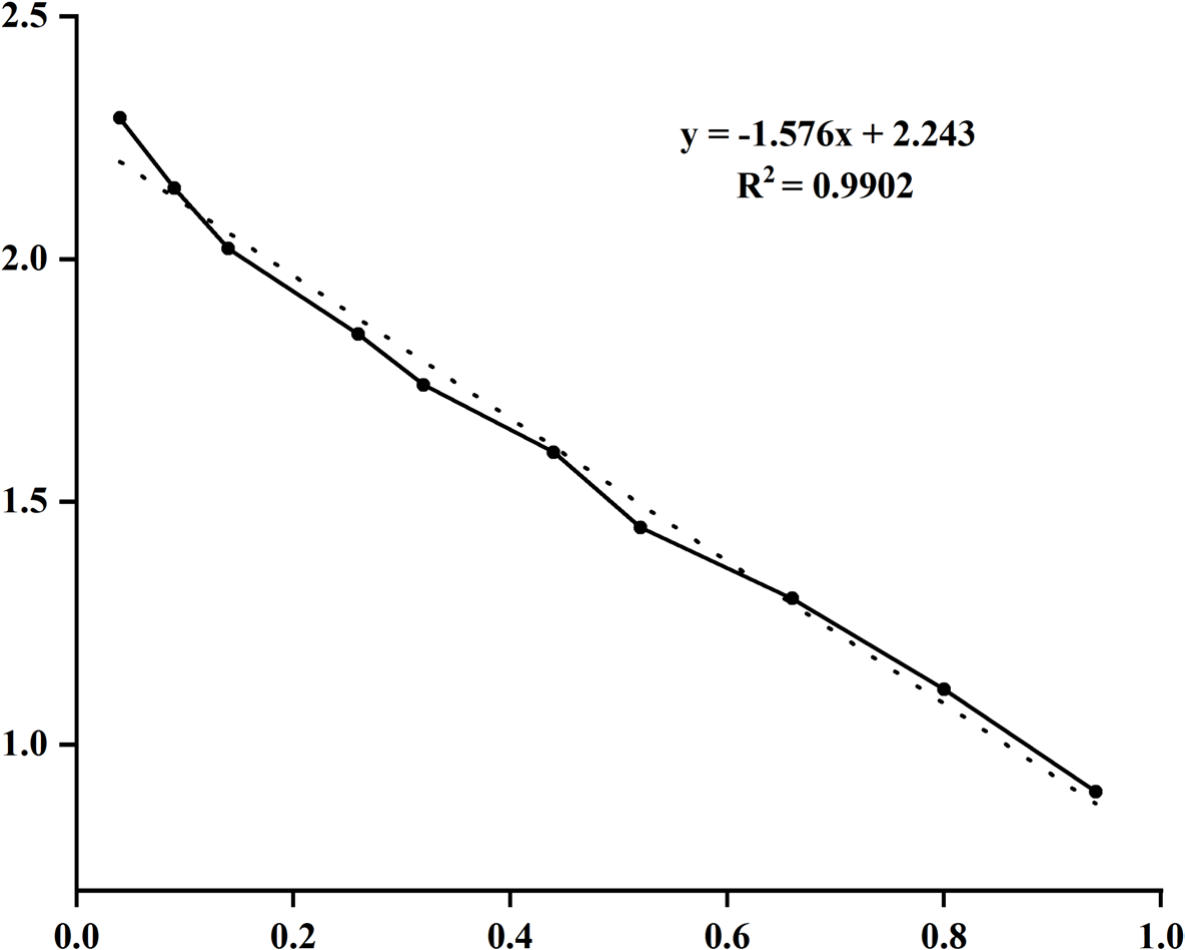
Standard curve. (X) standard sections with relative mobility; (Y) axis standard sections with corresponding to the relative molecular mass logarithmic

The standard curve, which determines the relative molecular mass of the protein, was derived from the data in Table 2 (Fig. 5). With a correlation coefficient *R*^*2*^ > 0.99, this suggests that the established standard curve is reliable for determining the relative molecular mass of the protein. The mobility of SNAP-25 in electrophoresis was determined to be 2.7. This value was incorporated into the standard curve equation for relative molecular mass determination, resulting in a calculated relative molecular mass for SNAP-25 of 29.3 kDa.

**Table 2:** Determination of protein band mobility in SNAP-25-SDS-PAGE

	**Measured Value**									
A/kDa	195	140	105	70	55	40	28	20	13	8
Rf	0.20	0.45	0.70	1.30	1.60	2.20	2.60	3.30	4.00	4.70
X	0.04	0.09	0.14	0.26	0.32	0.44	0.52	0.66	0.80	0.94
Y	2.29	2.14	2.02	1.84	1.74	1.60	1.44	1.30	1.11	0.90

*A *The relative molecular weight of each band of the standard substance,* Rf *Standard sections with the migration distance measurements, *X *Relative mobility of each band of the standard, *Y* Each band of the standard corresponds to the relative molecular mass log

**Fig. 6:**
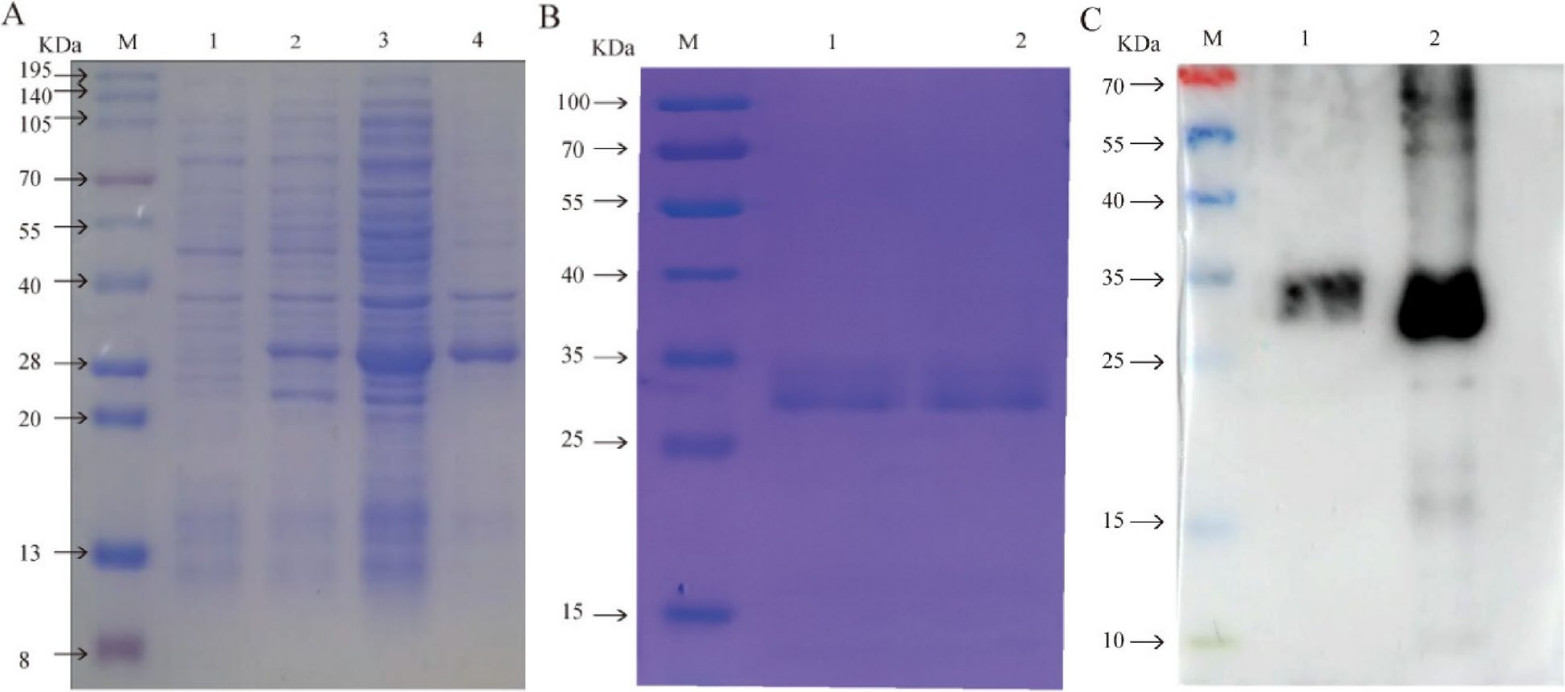
Prediction of sheep SNAP-25 recombinant protein expression form and WB results. **A **M: Protein molecular quality standard; 1: pre-induction products of recombinant bacteria; 2.: Recombinant bacteria induced products; 3: Supernatant of recombinant bacteria induced product; 4: Precipitation of recombinant bacteria-induced products; **B **M: Protein molecular quality standard; 1–2: Purified protein; **C **M: Protein molecular quality standard; 1: Tissue protein; 2: Purified protein

The recombinant strain was cultured at 37 °C until OD_600_ = 0.8, then induced with IPTG (1.0 mmol × L^−1^) for 6 h. Following ultrasonication, the bacteria were harvested, and both the supernatant and precipitate were isolated. SDS-PAGE analysis indicated that the post-induction product from the recombinant strain exhibited a distinct expression band in contrast to the pre-induction product. After sonication and centrifugation, the target band was evident in both the supernatant and precipitate of the induced recombinant bacterial product, indicating successful expression of recombinant SNAP-25 in BL21, aligning with expectations (Fig. 6A). Upon elution, a singular prominent band was observed, suggesting the protein underwent thorough purification (Fig. 6B).

The antibody titer was further verified by indirect ELISA. When the antiserum and the pre-immunized negative serum were diluted to 1:128,000 and 1:2000 separately, and with an OD450 (positive) to OD450 (negative) ratio ≥ 2.1, it confirmed an antibody serum titer of 1:128,000. Specific identification once again showed pronounced protein bands on the PVDF membrane, reinforcing the robust immunogenicity of the recombinant protein. The rabbit anti-sheep SNAP-25 antibody was found to bind selectively to both the recombinant and the endogenous protein (Fig. 6C).

### Effect of tapeworm *M. benedeni* infection on SNAP-25 expression in the small intestine of sheep

**Fig. 7:**
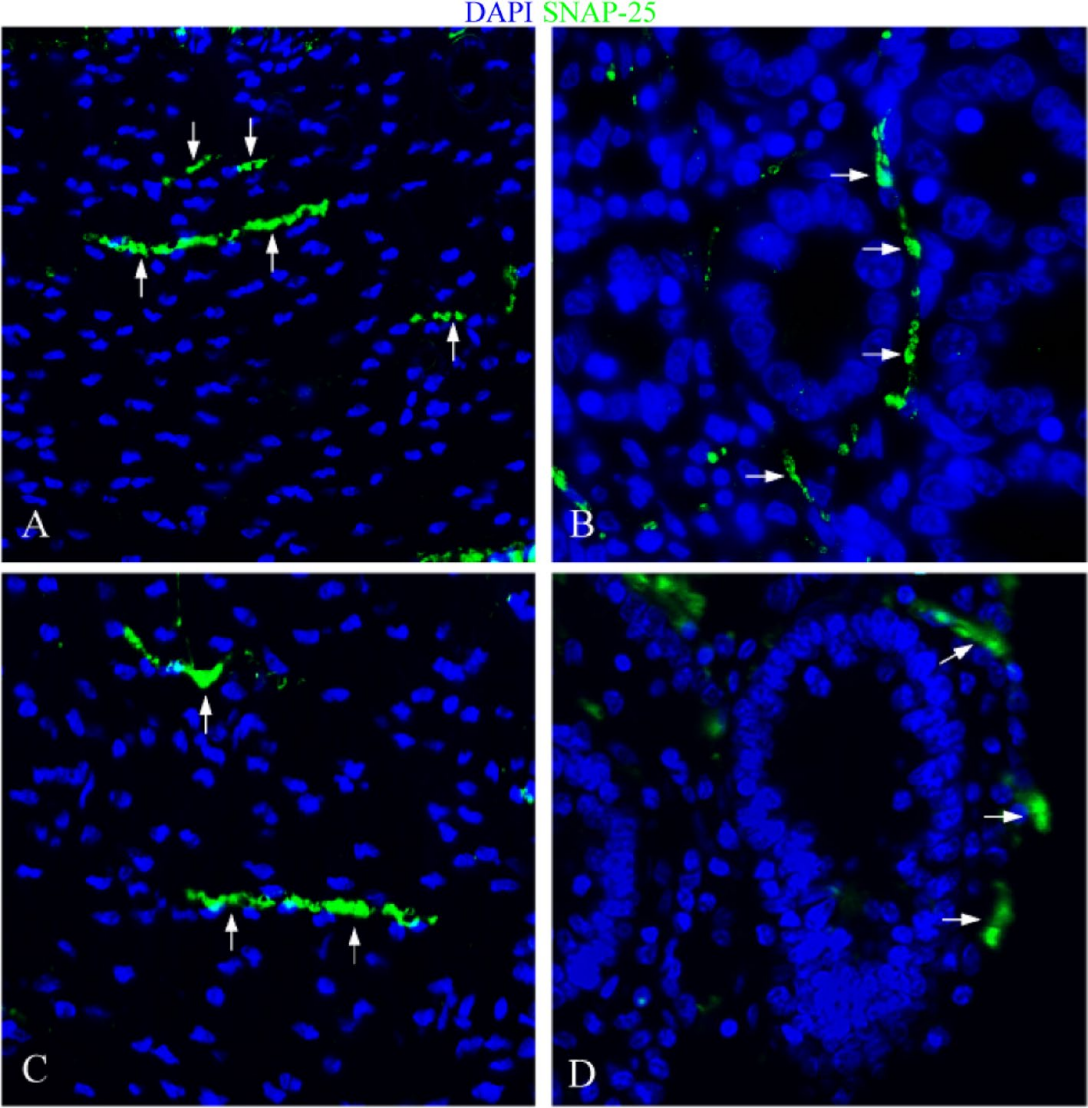
Distribution of SNAP-25 in duodenum. **A **The expression characteristics of SNAP-25 in muscle layer of control group; **B **The expression characteristics of SNAP-25 around the *lamina propria* intestinal glands in the control group; **C **The expression characteristics of SNAP-25 in muscle layer of infection group; **D **The expression characteristics of SNAP-25 around the *lamina propria* intestinal glands in the infection group. White arrows indicate nerve fibers; scale bar, 20 μm

**Fig. 8:**
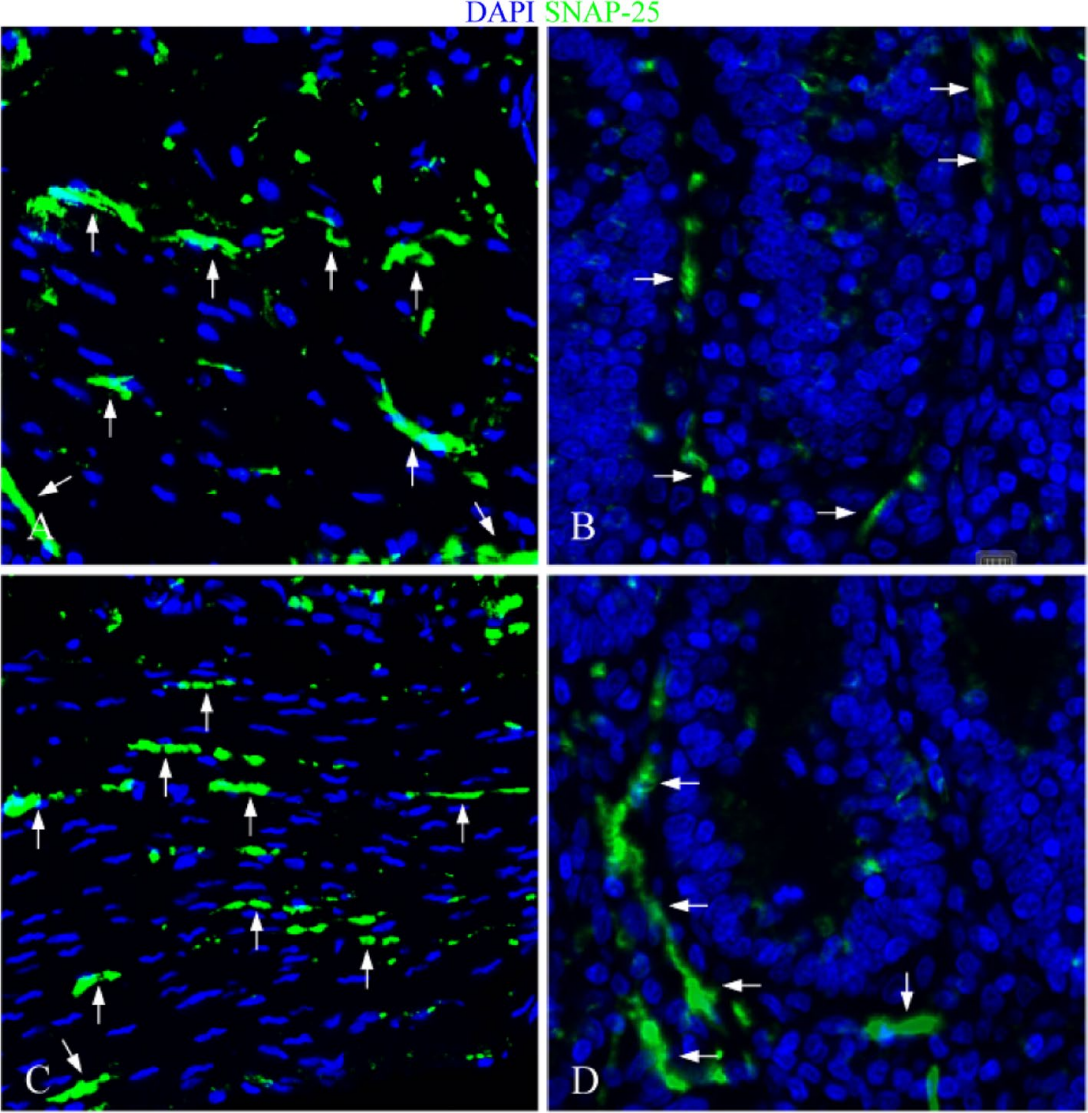
Distribution of SNAP-25 in jejunum. **A** The expression characteristics of SNAP-25 in muscle layer of control group; **B **The expression characteristics of SNAP-25 around the *lamina propria* intestinal glands in the control group; **C **The expression characteristics of SNAP-25 in muscle layer of infection group; **D** The expression characteristics of SNAP-25 around the *lamina propria* intestinal glands in the infection group. White arrows indicate nerve fibers; scale bar, 20 μm

**Fig. 9:**
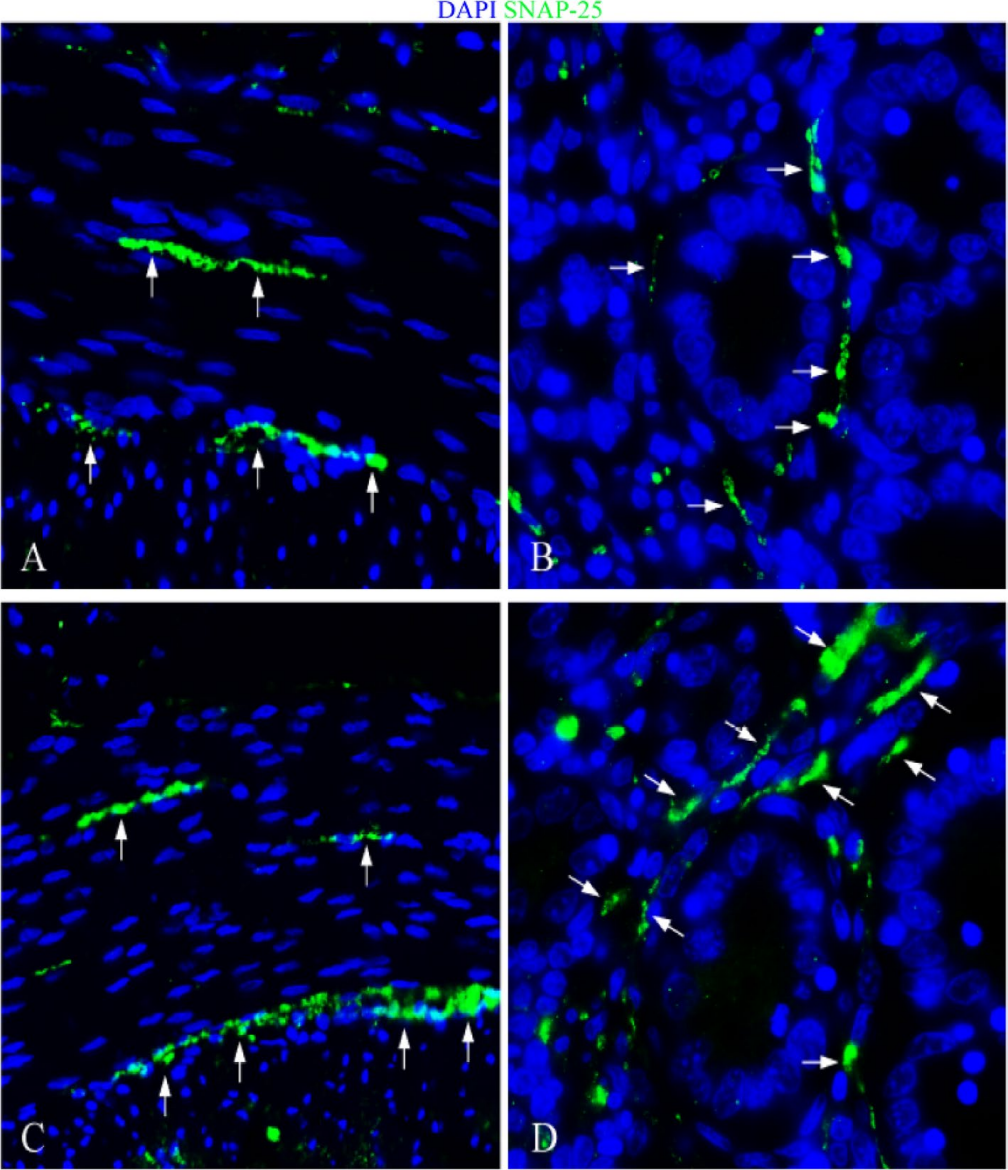
Distribution of SNAP-25 in ileum. **A **The expression characteristics of SNAP-25 in muscle layer of control group; **B **The expression characteristics of SNAP-25 around the *lamina propria* intestinal glands in the control group; **C **The expression characteristics of SNAP-25 in muscle layer of infection group; **D **The expression characteristics of SNAP-25 around the *lamina propria* intestinal glands in the infection group. White arrows indicate nerve fibers; scale bar, 20 μm

The experimental results indicated that in the control group, SNAP-25 was predominantly expressed on the nerve fibers surrounding the intestinal glands of the *lamina propria* (Fig. 7A, 8A, and 9A). It was also specifically expressed on the nerve fibers of the myenteric plexus (Fig. 7B, 8B, and 9B). In the infection group, the location of nerve fibers expressing SNAP-25 mirrored that of the control group. The protein was primarily expressed on the nerve fibers around the intestinal glands of the *lamina propria* and the nerve fibers of the myenteric plexus (Fig. 7C, 8C, 9C, 7D, 8D, and 9D).

**Fig. 10:**
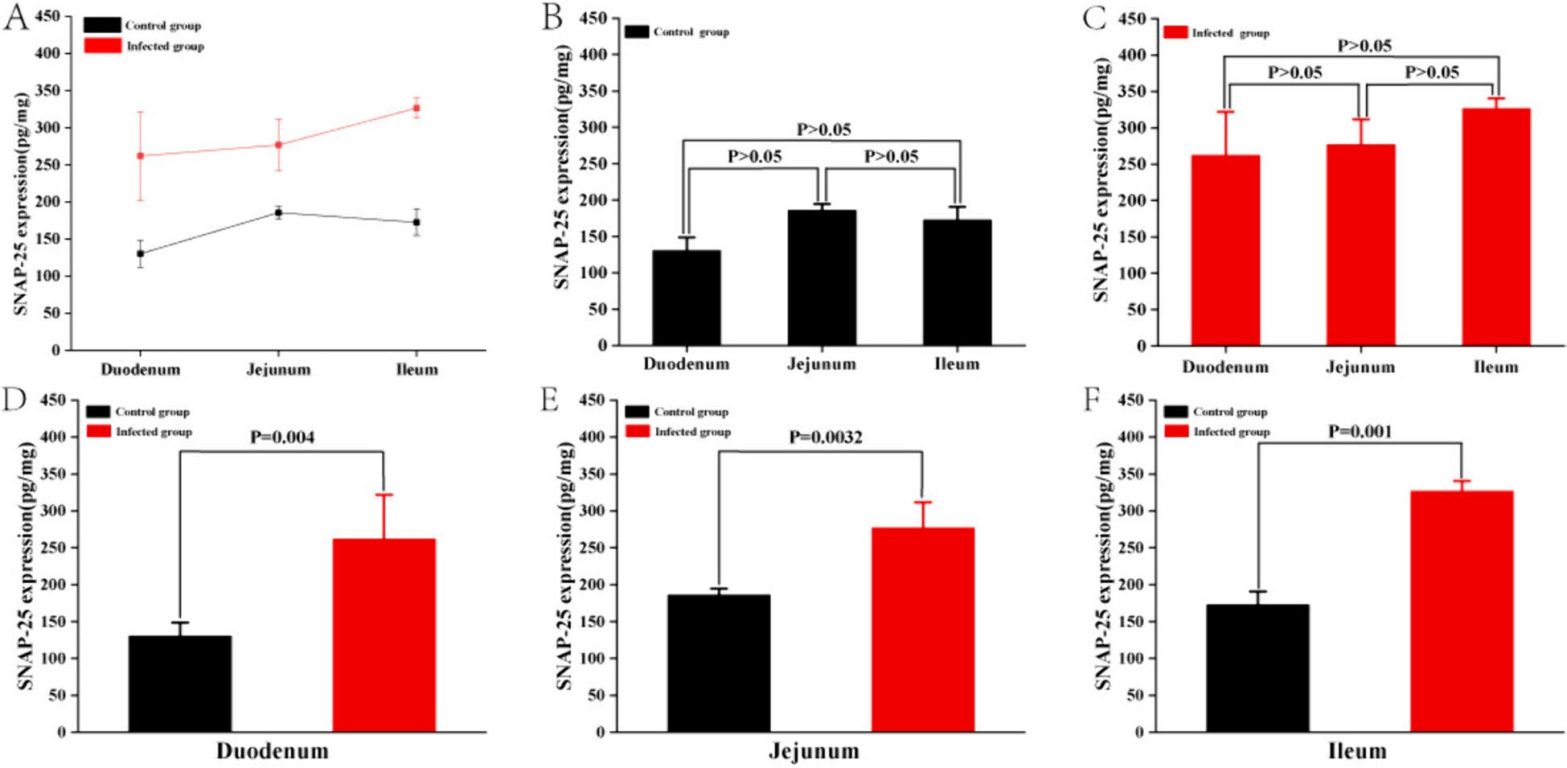
The expression of SNAP-25 in sheep small intestine

ELISA results demonstrated that the SNAP-25 content was lowest in the duodenum and highest in the jejunum of the control group (Fig. 10A). No significant difference was observed in the duodenum, jejunum, and ileum (*P* > 0.05) (Fig. 10B). Following *M. benedeni* infection, SNAP-25 expression increased progressively from the duodenum to the jejunum and then to the ileum (Fig. 10A). However, no significant disparity existed between the duodenum, jejunum, and ileum (*P* > 0.05) (Fig. 10C). The expression of SNAP-25 in the duodenum, jejunum, and ileum of the infected group exceeded that of the control group (*P* < 0.05) (Fig. 10D, E, and F). Furthermore, the SNAP-25 expression in the duodenum and ileum of the infected group was markedly greater than in the control group (*P* < 0.01) (Fig. 10D, F).

## Discussion

The results of bioinformatics analysis revealed that the CDs region gene of sheep SNAP-25 was 621 bp in length, encoding 206 amino acids, and the α-helix region of the SNAP-25 protein constitutes the largest portion, suggesting that the SNAP-25 protein possesses intricate biological functions. The tertiary structure predictions aligned with the findings of the secondary structure. This protein was identified as a stable, acidic, hydrophilic protein devoid of a signal peptide and transmembrane region, and the post-translational modification of SNAP-25 protein mainly involves phosphorylation. The SNAP-25 protein can interact with Vesicle Associated Membrane Protein II (VAMP2), Protruding Fusion Protein IA (STX1A), and others. SNAP-25 is referred to as a SNARE protein together with VAMP2 and STX1A. At present, it’s demonstrated that SNARE could form the “fusion pores” in all organisms containing nuclei (from single-celled organisms to animals, plants, and humans), allowing compounds to cross the cell or organelle biofilm, thus assisting in intercellular and intracellular communication [32]. Meanwhile, the secretion of mucin in the intestine of mice without VAMP was reduced, the intestinal barrier was damaged, and Treg cells and IL-10 were increased in the intestine of mice with VAMP knockout [33]. Syntaxin controls the release of mucin particles [34]. The rabbit anti-sheep SNAP-25 polyclonal antibody, synthesized via prokaryotic expression, has excellent specificity and is suitable for subsequent research. It has an important significance for further study of sheep response to *M. benedeni* infection.

The results showed that in the control group, nerve fibers specifically expressed SNAP-25 in the small intestine were abundantly distributed around the intestinal muscle layer and *lamina propria*. In particular, the expression levels in the jejunum were higher, the fluorescence signal was strongest, and the expression levels in the jejunum and ileum were higher than those in the duodenum. Studies have shown that SNAP-25 can be widely expressed in the central nervous system and peripheral nerves, and the digestive tract is also one of its high-expression sites. For example, SNAP-25 can be expressed in the gut of human [35–37], rats [8], mice [38, 39] and guinea pig [40]. In the human intestine, SNAP-25 is mainly expressed in the nerve fibers of *lamina propria* and muscle layer [35]. These research results are similar to those of current study. It suggests that SNAP-25 has the potential to be widely involved in the regulation of intestinal homeostasis in sheep.

Further analysis showed that *M. benedeni* infection did not change the spatial distribution of SNAP-25 nerve fibers, but significantly increased its expression levels in all intestinal segments (*P* < 0.05), especially in the duodenum and jejunum significantly increased (*P* < 0.01). SNAP-25 mainly promotes the release of neurotransmitters to exert its biological functions [41], such as promoting the release of norepinephrine [42] and neuropeptides [43], etc. Studies have found that β-adrenergic receptor (β_2_AR) distributed on the membrane of ILC2s in the small intestine can bind to norepinephrine neurotransmitters [44]. For example, it has been reported that the infection of *Clonorchis sinensis* can cause an increase of norepinephrine [45], which activates ILC2s after binding with β_2_AR, thus exerting the immune function related to anti-parasitic infection. Neuropeptide NMU mainly relies on two specific receptors, NMUR1 and NMUR2, to perform biological functions. Recent studies have shown that NMUR1 is expressed on ILC2s membrane in the *lamina propria* of the small intestine, and the expression of NMU can be significantly increased when *M. benedeni* infected [17], and ILC2s can be effectively activated by NMUR1 and act as the anti-parasitic infection functions [8, 46, 47]. The specific biological function of vasoactive intestinal peptide VIP depends on its two specific receptors VIPR1 and VIPR2. Previous studies have shown that intestinal ILC2s highly express VIPR1 [9], and VIP expression is significantly increased during fluke infection [48, 49] and ILC2s are activated by VIPR1. Then it plays the role of anti-inflammation and anti-parasite infection. Therefore, the results of this study suggest that *M. benedeni* infection leads to a significant increase in the expression of intestinal SNAP-25 in sheep intestines, which provides strong evidence for promoting intestinal nerve releases such as NMU, VIP and norepinephrine, and thus activating ILC2s as the core anti-parasitic immune response. It is also suggested that the intestinal neuroimmune regulatory network is one of the effective potential mechanisms in sensing and regulating *M. benedeni* infection.

## Conclusion

In this study, recombinant sheep SNAP-25 protein with a molecular weight of about 29.3 kDa was obtained based on prokaryotic expression technology, and rabbit anti-sheep SNAP-25 polyclonal antibody was successfully prepared, and it was proved that the antibody could specifically bind to the natural sheep SNAP-25 protein. Further analysis showed that the nerve fibers expressing SNAP-25 were mainly distributed around the intestinal gland and muscle layer of *lamina propria*, especially in the jejunum nerve fibers. *M. benedeni* infection did not change the spatial distribution characteristics of SNAP-25 nerve fibers, but its expression levels in all intestinal segments were significantly increased (*P* < 0.05), and there were significant differences in the duodenum and ileum (*P* < 0.01). The results of this study suggest that Monizia infection leads to a significant increase in the expression of intestinal SNAP-25 in sheep, which provides strong evidence for promoting intestinal nerve release such as NMU, VIP and norepinephrine, and thus activating ILC2s as the core anti-parasite immune response. It also suggests that the enteric neuroimmune regulatory network is one of the effective potential mechanisms for sensing and regulating Monizia infection.

## Materials and methods

### Experimental animals and experimental design

Twelve sheep were selected and categorized into two groups: a control group (*n* = 6, Control) and an infection group (*n* = 6, Infected), obtained from Wenkui Slaughterhouse, Liangzhou District, Wuwei City, Gansu Province. After intravenous anesthesia with pentobarbital sodium (20 mg/kg), the sheep were euthanized by carotid artery bloodletting. The abdominal cavity was then opened, and the small intestine, ranging from the stomach pylorus to the ileocecal orifice, was extracted. Following gentle rinsing of the intestinal food residue with sterilized normal saline, the duodenum, jejunum, and ileum tissues were swiftly excised. One sample was promptly placed in a 2.5 ml cryopreservation tube and stored in liquid nitrogen for subsequent analysis, while the other was immersed in a 4% neutral paraformaldehyde solution. These fixed tissue samples were then processed and sectioned using conventional methods to produce paraffin sections.

A healthy male New Zealand white rabbit, weighing approximately 2.5 kg, was procured from the Experimental Animal Center of the Lanzhou Veterinary Research Institute, which is affiliated with the Chinese Academy of Agricultural Sciences.

### Bioinformatics analysis

The physicochemical attributes, hydrophobicity, transmembrane structures, signal peptide regions, secondary and tertiary structures, glycosylation and phosphorylation sites, and protein interactions of the sheep SNAP-25 protein were comprehensively analyzed using various online prediction software (Table 3).

**Table 3:** Bioinformatics analysis software and website

Online Software	Website	Analysis /Forecast objects
ProtParam	https://web.expasy.org/protparam/	Protein physical and chemical properties
ProtScale	https://web.expasy.org/protscale/	Protein hydrophilic/hydrophobic
TMHMM2.0	https://services.healthtech.dtu.dk/services/TMHMM-2.0/	Transmembrane structures
SignalP-5.0	https://services.healthtech.dtu.dk/	Signal Peptide
PSIPRED 4.0	http://bioinf.cs.ucl.ac.uk/psipred	Secondary Structure
Swiss-Model	http://www.expasy.ch/swissmod/SWISS-MODEL.html	Three-level structure
NetPhos2.0	https://services.healthtech.dtu.dk/service.php?NetPhos-3.1	Phosphorylation site
NETOGlyc3.1	https://services.healthtech.dtu.dk/service.php?NetNGlyc-1.0	Glycosylation site
STRING	https://cn.string-db.org/	Protein interaction

### Preparation of polyclonal antibody against sheep SNAP-25

Based on the gene sequence of sheep SNAP-25 source from the NCBI database (NCBI Reference Sequence: XM_004014155.5), the coding region (CDs) was chosen for bioinformatics analysis. All identified amino acids were located in the extracellular region and lacked a signal peptide. After determining the enzyme digestion site, the sequence was dispatched to Genewiz Biotechnology Co., Ltd. for synthesis. This synthesized gene sequence was then ligated to the pET-28a ( +) vector and introduced into BL21 competent cells. Consequently, the positive recombinant plasmid pET-28a-SNAP-25, confirmed through correct plasmid sequencing, was acquired. The constructed pET-28a-SNAP-25 recombinant plasmid was introduced into competent BL21 (ED3) cells (sourced from Solarbio Biotechnology Co., Ltd.). A single colony was then inoculated into sterilized LB liquid medium (containing Kan^+^), and incubated at 37 °C until the OD_600_ measurement was between 0.6 and 0.8. Following a 1.0 mmol × L^−1^ IPTG induction over 6 h, the bacteria were harvested and ultrasonically lysed until clarity was achieved. Both supernatant and pellet were subsequently isolated, A nickel column containing His label was used to bind and purify the target protein. The protein content was determined by ultraviolet spectrophotometer.

The purified recombinant protein was mixed with the same amount of Freund’s complete adjuvant, and after fully emulsified, the rabbits were immunized by subcutaneous multi-points injection at back, scapular and popliteal lymph nodes at a dose of 800 μg per rabbit. One week later, the purified recombinant protein was fully emulsified with an equal amount of Freund’s incomplete adjuvant, and a dose of 400 μg per rabbit was subcutaneously injected at multiple points of back and scapular. After that, boost the immunization every one week (the method and dose were the same as the second time). Six days after the fourth immunization, blood was collected by heart punctures to obtain rabbit anti-sheep SNAP-25 poly-antiserum [50, 51].

### SNAP-25 polyclonal antibody titer detection and Western blotting analysis

The purified SNAP-25 recombinant protein was used as the antigen, added to the enzyme-linked immunosorbent assay (ELISA) plate (5 μg/well), and kept at 4℃ overnight. After being washed 3 times (5 min each time, the same below) with TBST, 5% skim milk powder was added to block at 37 °C for 1 h. After washing 3 times, the rabbit antiserum was diluted at a ratio of 1:2000, 1:4000, 1:8000, 1:16,000, 1:32,000, 1:64,000, and 1:128,000, respectively. The negative serum was diluted at a ratio of 1:2000. PBS was used as a blank control. 100 μL/well was added to the microplate in sequence and incubated at 37 °C for 1 h. Then, after washing 3 times, HRP-labeled goat anti-rabbit IgG (BOSTER Bioengineering Co., Ltd., 1:8000 dilution) was added, and incubated at 37 °C for 1 h. After washing again, TMB substrate chromogenic solution (Solarbio Biotechnology Co., Ltd.) was added in a dark environment, let stand for 15 min at room temperature 15 min of color development, 2 mol/L H_2_SO_4_ (50 μL/well) was added to stop the reaction. Finally, the absorbance value (OD) at 450 nm was measured with a microplate reader, and the highest dilution ratio of OD _(positive)_/OD _(negative)_ ≥ 2.1 was used as the titer of the multi-antibody serum [50].

According to the operation steps of the whole protein extraction kit (strong) (BC3710, Solarbio). The total protein was extracted from the normal sheep small intestine. The tissue protein and SNAP-25 recombinant protein were simultaneously denatured and subjected to SDS-PAGE gel electrophoresis (P1200, Solarbio) [52]. The proteins were transferred to PVDF membrane and blocked with 5% skim milk powder (37 °C, 2 h). Using rabbit antiserum as primary antibody (1:800) (4 °C, 12 h). Next, the membrane was washed three times with TBST and incubated with HRP-labeled goat anti-rabbit IgG (1:8000 dilution) for 2 h at room temperature. After washing 3 times with TBST again, ECL luminescent solution (Solarbio Biotechnology Co., Ltd.) was added dropwise for color development, exposed, and photographed [53].

### Expression and distribution characteristics of SNAP-25 in the small intestine of sheep

The tissue samples frozen at -80 °C were thawed on ice. A total of 1.0 g of tissue was accurately weighed, to which 1 ml of PBS and two magnetic beads were added. This mixture was then homogenized at -10 °C for 20 min and centrifuged for 10 min (4 °C, 12,000 rpm) to obtain the supernatant. The protein concentration was determined using the BCA Protein Assay Kit (Cat#PC0020, Lot No.20210908, Solarbio, Beijing, China). The SNAP-25 content in each segment of the intestinal tissue was assessed using ELISA (utilizing the Sheep SNAP-25 ELISA Kit from Shanghai enzyme-linked).

The paraffin sections prepared from the intestine (from both infection and control groups) underwent indirect immunofluorescence staining. The specific staining protocol is detailed as follows: Firstly, after deparaffinizing the sections and rehydrating them to water, they were immersed in boiling citrate antigen retrieval buffer, using a microwave set at 900W for 15 min. Upon natural cooling, the slides were rinsed in PBS (pH 7.4) on a decolorization shaker, repeated three times at 5 min each. Excess liquid was blotted off, and a perimeter was drawn around the tissue with a histochemical pen (to contain the reagents). Once the PBS dried, 5% BSA was applied dropwise, and the sections were incubated at 37 °C for 30 min. The blocking solution was carefully discarded, and the primary antibody (diluted 1:800) was introduced to the tissue. Slides were then stored in a humidity-controlled container and incubated at 4 °C overnight. Subsequently, they were washed in PBS (pH 7.4) on a decolorization shaker, repeated four times at 5 min intervals. Once partially dried, the secondary antibody (Goat Anti-Rabbit IgG H & L (Alexa Fluor ® 488) ab150077, abcam) was applied, ensuring the tissue was adequately covered, and then incubated at room temperature in the dark for 50 min. Slides were again washed in PBS (pH 7.4) on a decolorization shaker, repeated four times at 5 min each. Once the slides were partially dried, the DAPI staining solution was introduced and incubated at room temperature for 7 min. The slides were rinsed in PBS (pH 7.4) on a decolorization shaker, repeated three times for 5 min each. An autofluorescence quencher was applied for 5 min, followed by a 10 min water rinse. Once almost dry, the samples were mounted using an anti-fluorescence quenching medium. The spatial distribution of SNAP-25 in sheep's small intestines was observed under a fluorescent microscope and images were acquired (The DV Elite™ Imaging System, GE, USA). (DAPI: UV excitation wavelength 330–380 nm, emission 420 nm, blue; FITC: excitation 465–495 nm, emission 515–555 nm, green) [31].

### Statistical analysis

Data were presented in the form of Mean ± SD, and SPSS 23.0 (SPSS Inc., Chicago, USA) was used for statistical analysis. Differences between various parts within the same group were analyzed using a one-way analysis of variance (the LSD method was used for post hoc analysis), and the significant difference level was *P* < 0.05. Differences between identical parts of the infection group and the control group were assessed using an independent T-test, and the significant difference level was *P* < 0.05.

## Data Availability

No datasets were generated or analysed during the current study.

## Abbreviations

SNAP-25: Synaptosomal-associated Protein 25
*M.benedeni*: Moniezia benedeni
ILC2s: Group 2 innate lymphoid cells
ILC3s: Group 3 innate lymphoid cells
ELISA: Enzyme linked immunosorbent assay
NMU: Neuromedin U
VIP: Vasoactive intestinal peptide
SNARE: Soluble N-ethylmaleimide Sensitive Factor Attachment Protein Receptor
VAMP: Synaptic vesicle protein
Syntaxin: Synaptic fusion protein
β_2_AR: β_2_-Adrenergic receptor
